# High pro-neurotensin levels in individuals with type 1 diabetes associate with the development of cardiovascular risk factors at follow-up

**DOI:** 10.1007/s00592-021-01783-x

**Published:** 2021-08-29

**Authors:** Flavia Agata Cimini, Ilaria Barchetta, Laura Bertoccini, Valentina Ceccarelli, Marco Giorgio Baroni, Olle Melander, Maria Gisella Cavallo

**Affiliations:** 1 Department of Experimental Medicine, Sapienza University of Rome, Rome, Italy; 2 Department of Clinical Sciences Malmö, Lund University, Malmoe, Sweden; 3 Department of Internal Medicine, Skåne University Hospital, Malmö, Sweden; 4 Department of Clinical Medicine, Public Health, Life and Environmental Sciences (MeSVA), University of L’Aquila, L’Aquila, Italy; 5 Neuroendocrinology and Metabolic Diseases, IRCCS Neuromed, Pozzilli (Is), Italy

**Keywords:** Type 1 diabetes, Gastrointestinal peptides, Neurotensin, Cardiovascular disease, Biomarkers, Neuropeptides

## Abstract

**Aims:**

Neurotensin (NT) is a gut hormone that promotes lipids absorption and controls appetite. Elevated circulating pro-NT, the stable precursor of NT, is associated with cardiovascular (CV) disease, metabolic syndrome (MS) and type 2 diabetes (T2D). Features of MS and insulin resistance are reported also in type 1 diabetes (T1D), with detrimental impact on the overall CV risk profile. Aims of the study were to evaluate plasma pro-NT in T1D patients and to test whether its levels are associated with and/or predictive of CV risk factors and overall risk profile.

**Methods:**

For this longitudinal retrospective study, we analyzed clinical data from 41 T1D individuals referring to the diabetes outpatient clinics at Sapienza University of Rome, Italy, collected at the baseline and after 10 years. Fasting plasma pro-NT levels were measured in T1D subjects at the baseline and in 34 age-, sex-, BMI-comparable healthy individuals recruited in the same period.

**Results:**

Pro-NT did not differ significantly between patients and controls (median[range] pro-NT: 156.3 [96.6–198.2] vs. 179.4 [139.7–230.7] pmol/L, *p* = 0.26). In T1D, greater fasting pro-NT associated with poor glycemic control at baseline and predicted increased waist circumference, reduced insulin sensitivity, dyslipidemia and hypertension at 10-year follow-up. High pro-NT predicted 10-year very-high CV risk with adjusted OR = 11 (95%C.I.: 1.4–94.5; *p* = 0.029).

**Conclusions:**

In T1D individuals, elevated pro-NT levels predict the development of adverse metabolic profile, which translates in higher CV risk profile at 10-year follow-up. Pro-NT represents a novel predictor/marker of CV risk factors in adults with T1D.

## Introduction

Type 1 diabetes (T1D) is an organ-specific autoimmune disease characterized by the immune-mediated destruction of pancreatic *β*-cells, chronic hyperglycemia and the development of micro- and macro-vascular complications.

Individuals with T1D have an almost threefold higher mortality compared to the general population [1] largely due to premature cardiovascular (CV) disease [2, 3]. Data from randomized clinical trials showed that hyperglycemia is only one of the contributors of the high CV risk observed in these individuals [4–6]. Mounting evidence suggests an increasing occurrence of insulin resistance and related disorders with disease duration, such as systemic hypertension and altered lipid profile, with negative impact on diabetes’ complications [4–7].

Besides, several pathophysiological mechanisms, partially overlapping those occurring in type 2 diabetes (T2D) and metabolic syndrome (MS), have been demonstrated in T1D [6, 7]; among them, altered secretion pattern of gut peptide hormones, including amylin, ghrelin and glucose-dependent insulinotropic polypeptide (GIP), was found also in T1D individuals [8, 9].

Neurotensin (NT) is a gut hormone released by intestinal neuroendocrine cells in response to fat ingestion, facilitating fatty acids translocation through the intestinal mucosa [10–12]. NT also acts as a neurotransmitter by modulating the leptin-mediating food intake in the central nervous system [13, 14]. Moreover, NT is co-secreted with other gastrointestinal hormones, as GLP-1, and a direct influence on blood glucose control has been postulated [15].

Experimental data demonstrated that mice knocked-out for the NT gene are protected from the development of high fat diet-induced obesity, insulin resistance and hepato-steatosis [16]. In humans, higher circulating levels of pro-NT, the stable fragment of NT [17], have been associated with the presence and development of dysmetabolic conditions, such as T2D, obesity, non-alcoholic fatty liver disease (NAFLD), and with increased cardiovascular and all-cause mortality in large cohorts of adults [18–24]. Recently, our group demonstrated that high pro-NT levels are associated with bodyweight gain and the development of metabolic impairment in obese children [25].

Indeed, NT appears as a gut hormone entangled with processes regulating insulin resistance and glucose-lipid homeostasis that can lead to unfavorable CV risk profile.

So far, no data are available on pro-NT levels in individuals with T1D. Therefore, aims of this study were to assess circulating pro-NT concentration and its correlates in subjects with T1D, and to evaluate if pro-NT was a predictor of higher CV risk profile in this population at the 10-year follow-up.

## Research design and methods

### Study population

For this longitudinal study, we retrospectively analyzed data from forty-one individuals with T1D referring to the diabetes outpatient clinics at Sapienza University of Rome, Italy. We included individuals with a diagnosis of T1D, currently referring to our clinic for diabetes’ management and care and whom clinical data and frozen blood samples, collected 10 years earlier, were available.

In order to obtain reference values of fasting plasma pro-NT in the absence of diabetes and metabolic diseases, pro-NT levels were also measured in plasma samples from thirty-four age-, sex-, BMI-comparable healthy subjects (mean ± standard deviation (SD) age: 40.5 ± 10, sex (male/female): 16/18, BMI: 23.9 ± 2.8 kg/m^2^) recruited in the same period and selected as a control group for the basal evaluations.

For T1D subjects, we considered clinical data recorded in two different time points: at the baseline and after 10 years. Enrollment of the T1D cohort and controls was conducted between 2007 and 2009. Follow-up visits of T1D patients went from 2017 to 2019. Given the retrospective design of our study, data collected at the baseline and those recorded after a follow-up of 10 years were both available for all individuals in the T1D cohort.

### Clinical and biochemical evaluations

All study participants underwent complete work-up including clinical examination, anthropometric measurements and fasting blood sampling for routine biochemistry. Data on clinical and pharmacological history were also collected.

Weight, height and waist circumference were measured and body mass index calculated [BMI; weight (kg) x squared height (m^2^)]; systemic systolic (SBP) and diastolic (DBP) blood pressure were assessed after 5 min resting and mean values of three consecutive assessments were recorded.

Venous blood samples were collected after 12-h fasting for measuring blood glucose (FBG, mg/dL), glycosylated hemoglobin (HbA1c, %—mmol/mol), total cholesterol (mg/dL), high-density lipoprotein cholesterol (HDL, mg/dL), triglycerides (mg/dL), aspartate aminotransferase (AST, IU/L), alanine aminotransferase (ALT, IU/L), gamma glutamil transpeptidase (GGT, IU/L) and creatinine (mg/dL) by standards methods in the centralized lab.

Fasting serum insulin (FSI, IU/mL) was measured by radioimmunoassay (ADVIA Insulin Ready Pack 100; Bayer Diagnostics, Milan, Italy; intra- and inter-assay coefficients of variation < 5%).

Low-density lipoprotein cholesterol (LDL-C) value was obtained using Friedewald formula. Estimated glomerular filtration rate (eGFR, ml/min) was calculated by Cockcroft-Gault formula.

The insulin sensitivity was calculated by using the estimated insulin sensitivity (eIS) formula:

eIS = exp (4.1075−0.01299 [waist circumference, cm]−1.05819 [insulin dose, daily units per kg]−0.00354 [triglycerides, mg/dL]−0.00802 [DBP, mm Hg]) [26].

Diabetes mellitus was diagnosed according to the American Diabetes Association criteria [27].

MS was defined by the Consensus International Diabetes Federation and American Heart Association/National Heart, Lung, and Blood Institute 2009 [28].

CV risk was calculated according to the 2019 Guidelines on Diabetes, Pre-diabetes, and Cardiovascular Diseases of the European Society of Cardiology (ESC), developed in collaboration with the European Association of the Study of Diabetes (EASD) [29]. Based on these guidelines, patients with T1D are at: very high CV risk (10-year risk of CVD death > 10%) in the presence of established CV disease or early onset T1D of long duration (> 20 years); high risk.

(10-year risk of CV death 5−10%) in the presence of diabetes’ duration > 10 years without organ damage plus any additional risk factor; or at moderate risk in case of young patients (aged < 35 years) with T1D of short duration (< 10 years) [29].

### Pro-NT measurement

Fasting pro-NT levels were measured in both T1D and controls on plasma collected at the first time point observation (baseline). Plasma concentration of pro-NT, the stable NT precursor fragment released in equimolar amounts relative to NT, was measured on plasma collected after 12 h fasting, frozen immediately after separation and stored at −80 °C, using a chemiluminometric sandwich immunoassay to detect pro-NT amino acids 1–117, as previously described [25]. The limit of detection was 1.9 pmol/L. The mean inter assay coefficient of variability was 3.7% in the measuring range 3–270 pmol/L.

### Ethics standards

The study protocol was reviewed and approved by the local Ethics Committee and conducted in conformance with the Helsinki Declaration. Informed written consent was obtained from the participants before all the study procedures.

### Statistics

SPSS version 25.0 statistical package was used to perform the analyses. Values are shown as mean ± standard deviation (SD), median [interquartile range, IQR] or percentage, as appropriate. Variables with skewed distribution were log-transformed before the analyses. Differences between two independent groups were compared by Student's t-test for continuous variables and by *χ*^2^ test for categorical parameters, and comparisons between more than 2 groups were obtained by Bonferroni-adjusted ANOVA test. Correlations were estimated by Spearman’s test. Multivariate linear regression analyses were implemented to investigate the existence of an independent association between plasma pro-NT and HbA1c levels at the baseline, and to evaluate the association between basal pro-NT and eIS at the follow-up, after adjustments for sex, age and possible confounders measured at the baseline exam, as specified in the Results section for each model. *P* values < 0.05 were considered statistically significant with a confidence interval (C.I.) of 95%.

## Results

### Baseline data

Plasma pro-NT levels were comparable between T1D subjects and controls (pro-NT: 156.3 [96.6–198.2] vs. 179.4 [139.7–230.7] pmol/L, respectively, *p* = 0.26). Clinical characteristics of the T1D population and control group are summarized in Table 1.

**Table 1:** Clinical and biochemical characteristics of the type 1 diabetes study cohort at baseline and 10-year follow up and control group

	Type 1 diabetes group Baseline *n* = 41	Control group *n* = 34	Type 1 diabetes group Follow-up *n* = 41	*P* value^§^
Age (years)	36.5 ± 11.5	40.5 ± 10	46.5 ± 11.5	0.13
Gender (M%)	39	47	39	0.11*
BMI (Kg/m^2^)	24.4 ± 3.8	23.9 ± 2.8	24 ± 2.9	0.41
Waist circumference (cm)	82.4 ± 15.1	81.1 ± 12.4	86.6 ± 12.7	0.89
SBP (mmHg)	123 ± 9.1	118 ± 12	121.2 ± 9.5	0.07
DBP (mmHg)	77 ± 6.3	76 ± 9	77 ± 9.2	0.52
Total cholesterol (mg/dl)	180 ± 30	193 ± 40.7	187.7 ± 44.6	0.89
HDL-C (mg/dl)	61.5 ± 15	57.4 ± 15.5	62.5 ± 20.7	0.51
LDL-C (mg/dl)	82.8 ± 25.9	94.1 ± 33	107.2 ± 35.9	0.13
Triglycerides (mg/dl)	105.4 ± 33.7	80.6 ± 34.9	109.6 ± 59.1	0.09
FBG (mg/dl)	148.9 ± 69.2	88.5 ± 7.11	167.8 ± 62.9	< 0.001
HbA1c (%; mmol/mol)	7.2 ± 1.1; 55 ± 18	–	7.5 ± 0.78; 58 ± 15	–
Creatinine (mg/dl)	0.85 ± 0.2	0.5 ± 0.3	0.83 ± 0.16	0.28
eGFR (ml/min)	109.19 ± 31.5	111.3 ± 29.7	101.5 ± 22.1	0.41
AST (IU/I)	20.4 ± 9.9	19.6 ± 5.3	21.6 ± 7.6	0.72
ALT (IU/I)	19.1 ± 6.7	19.4 ± 4.5	24.3 ± 8.1	0.63
AST/ALT ratio	1.06 ± 0.35	1.01 ± 0.33	0.83 ± 0.29	0.56
GGT (IU/I)	15.2 ± 9.3	21.1 ± 10.5	18.7 ± 17.4	0.65
Median value [interquartile range] Pro-NT (pmol/L)	164.5 ± 96.6 156.3 [96.6–198.2]	186.4 ± 65.3 179.4 [139.7–230.7]	–	0.26
Duration of diabetes (years)	11.3 ± 10.6	–	21.5 ± 11	–
Statins therapy (%)	5	0	66	0.35*
Anti-hypertensive therapy (%)	2	0	68	0.49*
Metabolic syndrome (%)	2	0	66	0.49*
CV risk (moderate/high/very high, %)	66/30/4	–	13/31/56	–
Daily insulin dose (UI/day)	33.9 ± 29.2	–	40.2 ± 13.6	–
Insulin dose per kilogram (UI/kg)	0.47 ± 0.15	–	0.56 ± 0.2	–

Data are expressed as mean ± SD, unless otherwise indicated. §Comparison between subjects with type 1 diabetes at the baseline and controls (Student’s t-test or *χ^2^ test, as appropriate). *P* values < 0.05 are considered statistically significant

In T1D population, at the baseline, higher pro-NT levels were significantly associated with worse glycemic control, as indicated by greater FBG and HbA1c levels (*r* = 0.29, *p* = 0.05 and *r* = 0.4, *p* = 0.02, respectively), and a trend toward an association with triglycerides levels was observed, although it did not reach the statistical significance (*r* = 0.21, *p* = 0.07). No association was found between pro-NT and kidney function, as estimated by eGFR and serum creatinine levels (eGFR: *r* = 0.15, *p* = 0.41; serum creatinine: *r* = −0.06, *p* = 0.76).

At the linear multivariate analysis, the association between basal pro-NT and HbA1c levels was independent from age, sex and BMI (Standardized *β* = *p* = 0.035, R value of the model = 0.40).

### Follow-up analyses

Clinical characteristics of T1D population at the follow-up are summarized in Table 1.

Elevated pro-NT levels at the baseline significantly associated with greater waist circumference (*r* = 0.37, *p* = 0.01), daily insulin dose (*r* = 0.33, *p* = 0.01), insulin dose per kilogram (*r* = 0.55, *p* = 0.002), FBG (*r* = 0.58, *p* < 0.001), HbA1c (*r* = 0.45, *p* = 0.015) and triglycerides (*r* = 0.47, *p* = 0.011) at the 10-year follow-up examination.

Higher pro-NT at the baseline predicted reduced insulin sensitivity (i.e., eIS) at 10-year follow-up (*r* = −0.77, *p* < 0.001), and this association persisted significant after adjusting for sex, age, BMI and diabetes’ duration, all considered at the follow up (*p* = 0.018) (Table 2).

**Table 2:** Multivariate linear regression analysis

	Unstandardized		Standardized		
	*ß*	Standard Deviation Error	*ß*	*T*	*P* value
(Constant)	99.328	35.421		2.804	0.112
Pro-NT	− 0.136	0.053	− 0.526	− 2.583	0.018
Sex	4.238	8.325	0.090	0.509	0.616
Age follow-up	− 0.834	− 0.393	− 0.490	2.121	0.057
BMI follow-up	− 2.170	1.304	− 0.287	1.664	0.112
Duration of diabetes follow-up	− 0.478	0.582	− 0.200	− 0.821	− 0.421

Estimated insulin sensitivity (eIS) at 10-year follow-up is the dependent variable

*R* value of the model = 0.45

In addition, greater pro-NT levels at baseline were associated with the development of dyslipidemia (*r* = 0.72, *p* < 0.001), systemic hypertension (*r* = 0.53, *p* < 0.001), MS (*r* = 0.38, *p* = 0.017), and with an elevated CV risk (considered as an ordinal variable; *r* = 0.69, *p* < 0.001) after 10 years of follow-up.

**Fig. 1:**
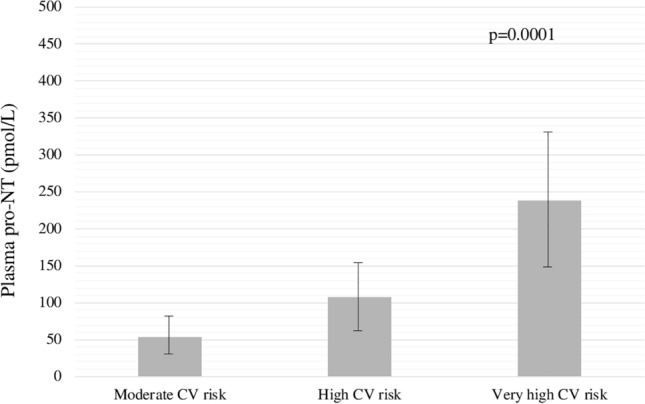
Plasma pro-NT levels at the baseline in relation to the presence of moderate, high, and very high CV risk at the 10-year follow-up. Bonferroni-adjusted ANOVA test

Pro-NT levels were significantly higher throughout increasing CV risk, as estimated by the ESC category [moderate risk: 54.2 ± 25.5 pmol/L, high risk: 107.2 ± 45.7 pmol/L, very high risk: 238.6 ± 90.4 pmol/L, *p* < 0.001, Fig. 1].

Individuals with pro-NT levels above the median value at baseline (*high-proNT;* median pro-NT in the T1D group = 161.8 pmol/L) showed significantly greater waist circumference (*p* = 0.03), LDL-cholesterol (*p* = 0.02), triglycerides (*p* = 0.01), FBG (*p* = 0.01), HbA1c (*p* = 0.04), insulin requirement (*p* = 0.04), and lower AST/ALT ratio (*p* = 0.02) at follow-up when compared with patients with basal pro-NT levels below the median value (*low-proNT)*.

After 10 years, the prevalence of dyslipidemia, hypertension, and MS was significantly higher in the *high-proNT* vs *low-proNT* group (all *p* < 0.001), putting almost the whole *high-proNT* cohort in the category at very high CV risk (Table 3).

**Table 3:** Clinical and biochemical characteristics of type 1 diabetes study cohort in relation to basal pro-NT levels (above and below the median value, *high-proNT* and *low-proNT,* respectively) at 10-year follow-up

	High-proNT subjects	Low-proNT subjects	*P* value
Age (years)	39.2 ± 11.2	33.8 ± 19.6	0.09
Gender (M%)	57%	65%	0.23*
BMI (Kg/m^2^)	24.07 ± 2.8	24.06 ± 3.1	0.93
Waist circumference (cm)	94.5 ± 14.6	80 ± 5.8	0.03
SBP (mmHg)	121.7 ± 9.7	120.6 ± 9.6	0.75
DBP (mmHg)	76.4 ± 9.2	77.6 ± 9.4	0.72
Total cholesterol (mg/dl)	181.7 ± 41.6	193.6 ± 48.3	0.49
HDL-C (mg/dl)	61.1 ± 24.6	63.8 ± 16.9	0.73
LDL-C (mg/dl)	115.5 ± 41.6	90.9 ± 28.4	0.02
Triglycerides (mg/dl)	107.4 ± 41	71.7 ± 28.3	0.01
FBG (mg/dl)	172.7 ± 53.2	124.5 ± 42.19	0.01
HbA1c (%; mmol/mol)	7.45 ± 0.79; 57 ± 15	6.86 ± 0.67; 51 ± 14	0.04
Creatinine (mg/dl)	0.81 ± 0.15	0.87 ± 0.17	0.27
eGFR (ml/min)	101.4 ± 22.4	99.5 ± 22.5	0.51
AST (IU/I)	21.7 ± 6.18	22.7 ± 15.9	0.38
ALT (IU/I)	26.1 ± 12.8	20.3 ± 8.1	0.04
AST/ALT ratio	0.83 ± 0.36	1.11 ± 0.24	0.02
GGT (IU/I)	16.5 ± 12.7	15.1 ± 5.3	0.11
Statins therapy (%)	100	33	0.001*
Anti-hypertensive therapy (%)	100	38	0.001*
Metabolic syndrome (%)	95	38	0.001*
CV risk (very high, %)	95	19	0.0001*
Daily insulin dose (UI/day)	45.4 ± 14.6	36.2 ± 11.5	0.04
Insulin dose per kilogram (UI/kg)	0.65 ± .0.21	0.47 ± 0.15	0.01

Data are expressed as mean ± SD, unless otherwise indicated. Differences have been compared by Student’s t-test or *χ^2^ test, as appropriate. *P* values < 0.05 are considered statistically significant

Finally, elevated pro-NT levels baseline predicted the development of very high CV risk after 10 years with an OR = 11 (95%C.I.: 1.4–94.5; *p* = 0.029) after adjustment for age, sex, diabetes’ duration and presence of metabolic syndrome, at the multivariate logistic regression analysis (*β* coefficient = 2.4, *R*^2^ of the model = 0.88).

## Conclusions

Our study demonstrates that in T1D patients, elevated plasma pro-NT levels associate with poor glycemic control and predict the development of abdominal adiposity and additional CV risk factors, such as hypertension and dyslipidemia, after 10-year follow up.

This is the first investigation conducted in T1D to explore circulating pro-NT in relation to metabolic profile and clinical outcomes. The association between higher pro-NT levels, obesity and impaired glucose metabolism has previously been demonstrated in both animal models [16, 30, 31] and in population-based epidemiological studies [18, 19, 22]. In our study, pro-NT range is comparable to that observed in larger populations, with and without T2D and other metabolic disorders [14, 19–22].

Our data show that higher basal pro-NT levels are associated with increased waist circumference in T1D subjects later in life, regardless of the bodyweight. As abdominal fat accumulation is an independent CV risk factor also in T1D [32, 33], the development of visceral adiposity may be one of the determinants of the higher CV risk observed in patients with T1D with high pro-NT levels at baseline.

The existence of a linear correlation between pro-NT and BMI was demonstrated in previous reports and was mainly mediated by an underlying condition of insulin resistance [16].

Accordingly, in our T1D cohort, higher basal pro-NT correlated with greater daily insulin dose and insulin dose per kilogram and associated with reduced insulin sensitivity at follow-up independently from confounders.

In animal models, experimentally induced loss of NT gene translates in less pronounced diet-induced insulin resistance in comparison with wild type [16, 34]. Protection from insulin resistance was also displayed by rodents lacking the NT receptor NTSR3 [34], which is directly implicated in the modulation of the glucose transporter GLUT4, one of the major regulators of insulin sensitivity in adipose tissue.

Our group and other investigators reported that high pro-NT is associated with the presence of NAFLD and metabolic diseases in adults [18, 20–24] and predicted bodyweight gain and impaired glucose–insulin metabolism in obese children [25]. We also observed high circulating pro-NT levels in obese individuals with adipose tissue inflammation and dysfunction [19], conditions strictly associated with insulin resistance [35]. Recent data from the REasons for Geographic and Racial Differences in Stroke (REGARDS) study show that higher pro-NT at the baseline predicts the onset of metabolic syndrome, and in particular low HDL levels and impaired glucose regulation, in a large cohort of over 3700 participants, independent of demographical factors [36].

Thus, the overall negative impact of NT on insulin sensitivity may be at the basis of the association between higher pro-NT, FBG and HbA1c observed in our study and may explain—at least in part—the development of a more pronounced dysmetabolic trait in T1D individuals with high-NT levels at baseline.

At follow-up, T1D individuals with high pro-NT displayed greater amount of abdominal fat, LDL-cholesterol and triglycerides, worse glycemic control, increased insulin requirement, and lower AST/ALT ratio—suggestive for hepatic fat accumulation—in comparison to those with low-NT. Overall, these alterations resulted in higher prevalence of dyslipidemia, hypertension and MS later in life in high-proNT versus low-proNT groups.

NT secretion increases in the post-prandial state in relation to food fat content [11], and the magnitude of NT secretion is proportional to the extension of post-prandial hyper-triglycaeridemia [16, 21]. Consequently, we might speculate that if higher pro-NT is associated, in the short run, to greater blood lipid levels after a fatty meal, in the long term this condition may induce increased adipose tissue fat content, free fatty acids release, possibly lipotoxicity, and may predispose to insulin resistance.

Many investigations reported in T1D patients an increased prevalence of features commonly associated with T2D, reflecting impaired insulin sensitivity, along with the classical manifestation of T1D, leading to the need of identifying a third phenotype of diabetes *in-between*, the so-called ‘double-diabetes’ [7, 37–39].

Individuals with double-diabetes may present traditional CV risk factors, i.e., overweight, hypertension and dyslipidemia that act synergistically with chronic hyperglycemia and worsen the CV risk profile, as demonstrated in large prospective studies in type 1 diabetes populations [4–7, 40].

Data from the FinnDiane study showed that in T1D subjects, the phenotype associated with the highest CV mortality is the one characterized by a combination of poor glycemic control, atherogenic lipid profile, central obesity and insulin resistance [40].

The identification of early markers of susceptibility to cardio-metabolic risk factors later in life may be of clinical relevance in T1D management and care. In this study, we demonstrated the association between basal plasma pro-NT levels and the development of worse CV risk profile at the 10-year follow up in T1D individuals, despite an acceptable glycemic control (mean HbA1c at follow-up: 7.5%) and independently from potential confounders such as sex, age, diabetes’ duration and the presence of metabolic syndrome.

This study has some limitations. First, the retrospective design may provide inferior level of evidence compared to prospective design and may expose to selection bias. Moreover, in this study, the level of insulin sensitivity has been estimated by eIS, rather than directly assessed by gold-standard techniques as the euglycemic hyperinsulinemic clamp. However, despite its undoubtable role in research, this test finds scarce applicability in the clinical setting, as it represents an invasive, costly, time and personnel consuming procedure. Indeed, the eIS is a commonly used estimator of overall insulin sensitivity, which has been validated with the euglycemic hyperinsulinemic clamp in patients with T1D [26]. Finally, this is an exploratory study on the relationship between pro-NT and T1D; therefore, further studies with a larger sample size are warranted in order to confirm our study findings. Some recent investigations found the association between higher pro-NT levels and impaired renal function, likely reflecting reduced pro-NT excretion in the presence of kidney disease [41]. Contrariwise, in our study, no correlation was shown between pro-NT and the eGFR. However, no study participant had acute/chronic kidney disease or diabetic nephropathy; therefore, a major role of renal function in influencing pro-NT levels in this study population was not expected.

Mechanisms behind the relationship between pro-NT and high CV risk remain partially unidentified. Based on ours and previous data, it can be speculated that NT contributes to the development of accumulating cardiometabolic risk factors by influencing food intake [14–16] and overall energy balance, though increased intestinal lipid absorption [11, 12, 21], altered glucose metabolism [15, 16, 18, 22], adipose tissue homeostasis [19] and body fat distribution [22].

In conclusion, this study demonstrates for the first time that elevated levels of fasting pro-NT are associated with the development of cardiovascular risk factors in patients with T1D, resulting in worse calculated CV risk profile, despite an overall acceptable glucose control. Our findings add insights to potential pathways behind metabolic alterations observed in T1D and point toward a potential role of pro-NT as a novel biomarker for CV risk stratification also in this population.

## Declarations

### Data availability

The data analyzed during the current study are available from the corresponding author on request.

## References

[1] Patterson CC, Karuranga S, Salpea P (2019). Worldwide estimates of incidence, prevalence and mortality of type 1 diabetes in children and adolescents: results from the International Diabetes federation diabetes atlas. Diabetes Res Clin Pract.

[2] Lee YB, Han K, Kim B, Lee S-E, Jun JE, Ahn J (2019). Risk of early mortality and cardiovascular disease in type 1 diabetes: a comparison with type 2 diabetes, a nationwide study. Cardiovasc Diabetol.

[3] Rawshani A, Sattar N, Franzen S, Rawshani A, Hattersley AT, Svensson AM (2018). Excess mortality and cardiovascular disease in young adults with type 1 diabetes in relation to age at onset: a nationwide, register-based cohort study. Lancet.

[4] Orchard TJ, Olson JC, Erbey JR (2003). Insulin resistance-related factors, but not glycemia, predict coronary artery disease in type 1 diabetes: 10-year follow-up data from the pittsburgh epidemiology of diabetes complications study. Diabetes Care.

[5] Pané A, Conget I, Boswell L, Ruiz S, Viñals C, Perea V, Giménez M, Cofán M, Blanco J, Vinagre I, Esmatjes E, Ortega E, Amor AJ (2020). Insulin resistance is associated with preclinical carotid atherosclerosis in patients with type 1 diabetes. Diabetes Metab Res Rev.

[6] Thorn LM, Forsblom C, Fagerudd J, Thomas MC, Pettersson-Fernholm K, Saraheimo M, Wadén J, Rönnback M, Rosengård-Bärlund M, Björkesten CG, Taskinen MR, Groop PH, FinnDiane Study Group (2005). Metabolic syndrome in type 1 diabetes: association with diabetic nephropathy and glycemic control (the FinnDiane study). Diabetes Care.

[7] Kilpatrick ES, Rigby AS, Atkin SL (2007). Insulin resistance, the metabolic syndrome, and complication risk in type 1 diabetes: "double diabetes" in the diabetes control and complications trial. Diabetes Care.

[8] El-Salhy M (1999). Neuroendocrine peptides in stomach and colon of an animal model for human diabetes type I. J Diabetes Complic.

[9] Huml M, Kobr J, Siala K (2011). Gut peptide hormones and pediatric type 1 diabetes mellitus. Physiol Res.

[10] Goedert M, Emson PC (1983). The regional distribution of neurotensin-like immunoreactivity in central and peripheral tissues of the cat. Brain Res.

[11] Ferris CF, Hammer RA, Leeman SE (1981). Elevation of plasma neurotensin during lipid perfusion of rat small intestine. Peptides.

[12] Piatek J, Witmanowski H, Paluszak J, Krauss H, Krawczyk J (2005). The effects of neurotensin on selected parameters of lipid metabolism in rats. Peptides.

[13] Brown JA, Bugescu R, Mayer TA (2017). Loss of action via neurotensin-leptin receptor neurons disrupts leptin and ghrelin-mediated control of energy balance. Endocrinology.

[14] Barchetta I, Ciccarelli G, Cimini FA (2018). Association between systemic leptin and neurotensin concentration in adult individuals with and without type 2 diabetes mellitus. J Endocrinol Invest.

[15] Grunddal KV, Ratner CF, Svendsen B (2016). Neurotensin is coexpressed, coreleased, and acts together with GLP-1 and PYY in enteroendocrine control of metabolism. Endocrinology.

[16] Li J, Song J, Zaytseva YY (2016). An obligatory role for neurotensin in high-fat-diet-induced obesity. Nature.

[17] Ernst A, Hellmich S, Bergmann A (2006). Proneurotensin 1–117, a stable neurotensin precursor fragment identified in human circulation. Peptides.

[18] Melander O, Maisel AS, Almgren P (2012). Plasma proneurotensin and incidence of diabetes, cardiovascular disease, breast cancer, and mortality. JAMA.

[19] Barchetta I, Cimini FA, Capoccia D (2018). Neurotensin is a lipid-induced gastrointestinal peptide associated with visceral adipose tissue inflammation in obesity. Nutrients.

[20] Fawad A, Bergmann A, Struck J, Nilsson PM, Orho-Melander M, Melander O (2018). Proneurotensin predicts cardiovascular disease in an elderly population. J Clin Endocrinol Metab.

[21] Fawad A, Fernandez C, Bergmann A (2020). Magnitude of rise in proneurotensin is related to amount of triglyceride appearance in blood after standardized oral intake of both saturated and unsaturated fat. Lipids Health Dis.

[22] Barchetta I, Cimini FA, Leonetti F (2018). Increased plasma proneurotensin levels identify NAFLD in adults with and without type 2 diabetes. J Clin Endocrinol Metab.

[23] Januzzi JL, Lyass A, Liu Y (2016). Circulating proneurotensin concentrations and cardiovascular disease events in the community: the framingham heart study. Arterioscler Thromb Vasc Biol.

[24] Wettersten N, Cushman M, Howard VJ (2018). Usefulness of proneurotensin to predict cardiovascular and all-cause mortality in a United States population (from the reasons for geographic and racial differences in stroke study). Am J Cardiol.

[25] Barchetta I, Bertoccini L, Sentinelli F (2020). Circulating pro-neurotensin levels predict bodyweight gain and metabolic alterations in children. NMCD.

[26] Duca LM, Maahs DM, Schauer IE (2016). Development and validation of a method to estimate insulin sensitivity in patients with and without type 1 diabetes. J Clin Endocrinol Metab.

[27] Standards of Medical Care in Diabetes—2021 Diabetes Care Jan 2021, 44 (Supplement 1) S1-S2; DOI: 10.2337/dc21-Sint 10.2337/dc21-Sint 33298409

[28] Alberti KG, Eckel RH, Grundy SM, Zimmet PZ, Cleeman JI, Donato KA (2009). Harmonizing the metabolic syndrome: a joint interim statement of the International diabetes federation task force on epidemiology and prevention; national heart, lung, and blood institute; american heart association; world heart federation; international atherosclerosis society; and international association for the study of obesity. Circulation.

[29] Cosentino F, Grant PJ, Aboyans V (2020). 2019 ESC guidelines on diabetes, pre-diabetes, and cardiovascular diseases developed in collaboration with the EASD. Eur Heart J.

[30] Saiyasit N, Chunchai T, Apaijai N (2020). Chronic high-fat diet consumption induces an alteration in plasma/brain neurotensin signaling, metabolic disturbance, systemic inflammation/oxidative stress, brain apoptosis, and dendritic spine loss. Neuropeptides.

[31] Rabinowich L, Fishman S, Hubel E (2015). Sortilin deficiency improves the metabolic phenotype and reduces hepatic steatosis of mice subjected to diet-induced obesity. J Hepatol.

[32] Patel P, Abate N (2013). Body fat distribution and insulin resistance. Nutrients.

[33] Williams KV, Erbey JR, Becker D, Arslanian S, Orchard TJ (2000). Can clinical factors estimate insulin resistance in type 1 diabetes?. Diabetes.

[34] Morris NJ, Ross SA, Lane WS (1998). Sortilin is the major 110-kDa protein in GLUT4 vesicles from adipocytes. J Biol Chem.

[35] Cimini FA, Barchetta I, Ciccarelli G (2021). Adipose tissue remodelling in obese subjects is a determinant of presence and severity of fatty liver disease. Diabetes Metab Res Rev.

[36] Nicoli CD, Carson AP, Plante TB, Long DL, McClure LA, Schulte J, Cushman M (2021). Pro-Neurotensin/Neuromedin N and Risk of Incident Metabolic Syndrome and Diabetes Mellitus in the REGARDS Cohort. J Clin Endocrinol Metab.

[37] Teupe B, Bergis K (1991). Epidemiological evidence for ‘‘double diabetes’’. Lancet.

[38] Cleland SJ (2012). Cardiovascular risk in double diabetes mellitus – when two worlds collide. Nat Rev Endocrinol.

[39] Merger SR, Kerner W, Stadler M, Zeyfang A, Jehle P, Muller-Korbsch M (2016). Prevalence and comorbidities of double diabetes. Diabetes Res Clin Pract.

[40] Makinen VP (2008). Metabolic phenotypes, vascular complications, and premature deaths in a population of 4,197 patients with type 1 diabetes. Diabetes.

[41] Tönjes A, Hoffmann A, Kralisch S (2020). Pro-neurotensin depends on renal function and is related to all-cause mortality in chronic kidney disease. Eur J Endocrinol.

